# Modulation of connexin 43 in viral infections

**DOI:** 10.1016/j.tvr.2024.200296

**Published:** 2024-11-08

**Authors:** Harry Scott, Patricia E. Martin, Sheila V. Graham

**Affiliations:** a MRC-University of Glasgow Centre for Virus Research, School of Infection and Immunity, College of Medical Veterinary and Life Sciences, University of Glasgow, Garscube Estate, Glasgow, G61 1QH, UK; b Department of Biological and Biomedical Science, School of Health and Life Sciences, Glasgow Caledonian University, Glasgow, G4 0BA, UK

**Keywords:** Connexin 43, Gap junctions, Hemichannels, Human papillomavirus, Human adenovirus type 5, Human immunodeficiency virus, Severe acute respiratory syndrome coronavirus 2

## Abstract

Connexins are essential for intercellular communication through gap junctions and the maintenance of cellular and tissue homeostasis. Connexin 43 (Cx43) is the most ubiquitously expressed connexin. As well as regulating homeostasis, Cx43 hemichannels and gap junctions play important roles in inflammation and the immune response. This, coupled with a range of non-channel functions performed by Cx43 makes it an attractive target for viruses. Recently, several groups have begun to explore the relationship between Cx43 and viral infection, with a diverse array of viruses being found to alter Cx43 hemichannels/gap junctions. Importantly, this includes several small DNA tumour viruses, which may target Cx43 to promote tumorigenesis. This review focuses on the ability of selected RNA/DNA viruses and retroviruses to either positively or negatively regulate Cx43 hemichannels and gap junctions in order to carry out their lifecycles. The role of Cx43 regulation by tumour viruses is also discussed in relation to tumour progression.

## Abbreviations

ACE2: Angiotensin converting enzyme 2
AGA: α-glycyrrhetinic acid
AIDS: Acquired immunodeficiency syndrome
AKT: Protein kinase B
APC: Adenomatous polyposis coli
cGAMP: cyclic GMP-AMP
cGAS: cyclic GMP-AMP synthase
CIN: Cervical intraepithelial neoplasia
CK1: Casein kinase 1
Cx43: Connexin 43
DAMP: Damage associated molecular pattern
DAPI: 4′,6-diamidino-2-phenylindole
Dlg1: human Discs large homologue 1
E: Envelope protein
E6: Early protein 6
GJIC: Gap junctional intercellular communication
HAdV-5: Human adenovirus type 5
HAND: HIV associated neurocognitive disorder
HHV8: Human herpesvirus 8
HIV: Human immunodeficiency virus
HPV: Human papillomavirus
IE: Immediate early
L: Late protein
LCR: Long control region
M: Membrane protein
MAGUK: Membrane associated guanlyate kinase
MCC: Merkel cell carcinoma
MCPyV: Merkel cell polyomavirus
N: Nucleocapsid protein
NIKS: Normal immortalised keratinocytes
PAMP: Pathogen associated molecular pattern
PDZ: Post-synaptic density protein of 95kDa/Drosophila Discs large tumour suppressor/Zonula occludens-1
PKA: Protein kinase A
PKC: Protein kinase C
PTEN: Phosphatase and tensin homologue deleted on chromosome 10
RIG-1: Retinoic acid-inducible gene 1
S: Spike protein
SARS-CoV-2: Severe acute respiratory syndrome coronavirus 2
STING: Stimulator of interferon genes
SV40: Simian virus 40
TNT: Tunneling nanotube
ZO-1: Zonula occludens-1

## 1 Introduction

**Fig. 1:**
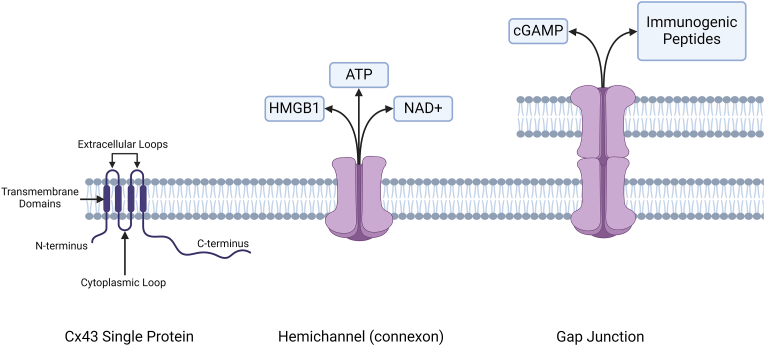
**Structure of an individual Cx43 protein, a hemichannel (connexon) and a gap junction channel, with factors involved in inflammation and the immune response shown.** Cx43 contains four α-helical transmembrane domains, two extracellular loops, a cytoplasmic loop and cytoplasmic N- and C-termini. A connexon is formed of six connexin proteins, which can be either homomeric or heteromeric depending on individual connexin protein compatibility [4]. Individual connexons inserted into the plasma membrane can act as hemichannels to release certain factors into the extracellular environment, including molecules involved in inflammation such as HMGB1, ATP and NAD+. Docking of two opposing connexons on the surfaces of different cells causes formation of a fully functional gap junction channel. These channels can also share factors involved in the immune response such as cGAMP for activation of the cGAS/STING pathway in adjacent cells and immunogenic peptides which can be presented on the surface of cells for recognition and killing of infected cells/cells adjacent to infected cells. *Created in BioRender. Scott, H. (2024) BioRender.com/x05r178*.

Viral infection is known to alter almost all aspects of cellular function, primarily to evade the host immune response and promote efficient replication and spread of the infecting virus to other cells. Intercellular communication represents both a help and a hindrance to these aims, as it can be hijacked to promote sharing of factors between cells to enhance spread of infection but is also instrumental in activation of the immune response [1]. Therefore, many viruses have evolved factors to modulate cellular communication, for example by producing proteins that antagonise the ability of infected cells to produce antigens that could then be recognised by the immune system and lead to an immune response [2]. One major component of cell-cell signalling that can be targeted by viruses of vertebrates is gap junctional intercellular communication (GJIC). Gap junctions allow direct communication between adjacent cells by the creation of intercellular channels. These gap junction channels, which are composed of transmembrane connexin proteins, allow the exchange of factors <1 kDa in size such as ions, metabolites and secondary messengers between cells and are essential for maintaining cellular homeostasis [3]. Gap junction channels are formed through oligomerisation of six individual connexin proteins to produce a connexon, which upon insertion into the plasma membrane can dock with an opposing connexon on an adjacent cell to create a gap junction channel (Fig. 1) [4].

Individual connexons are additionally capable of acting as hemichannels when localised to the plasma membrane, where they can release factors such as ATP and NAD+ into the extracellular environment [5]. These hemichannels differ in function from gap junctions, as they are involved in autocrine and paracrine signalling rather than intercellular communication. Unlike gap junctions, hemichannels mainly remain closed under physiological conditions and are instead opened during cell stress and inflammation, such as during infection with viruses or other pathogens [6].

Compared to most transmembrane proteins, connexins have relatively short-half lives of around 1–4h and are dynamically regulated by many different factors at the transcriptional, translational and post-translational levels [[7], [8], [9]]. This means that connexin expression and gap junctional communication can be altered rapidly in response to changes in the environment or during different stages of cellular processes such as cell proliferation [10,11].

The connexin protein family is made up of 21 members in humans, with connexin 43 (Cx43, encoded by the *GJA1* gene) being the most well-documented. This is partially due to Cx43 being the most widely-expressed of the connexins, with the protein being present in a diverse array of cells including cardiomyocytes [12], neurons [13] and epithelial cells [14], including keratinocytes [15]. In addition, the long cytoplasmic C-terminal tail of Cx43 is a source of interest as it allows interaction with a range of cellular proteins. These include proteins involved in regulating the lifecycle of Cx43 such as Zonula Occludens-1 (ZO-1) and human Discs large homologue 1 (Dlg1) [[16], [17], [18]], as well as proteins with roles in intracellular signalling pathways like β-catenin and cyclin E [[19], [20], [21]]. This ability of Cx43 to interact with proteins involved in other pathways enables Cx43 to directly influence processes other than intercellular communication, such as cellular proliferation and apoptosis [22,23].

**Fig. 2:**
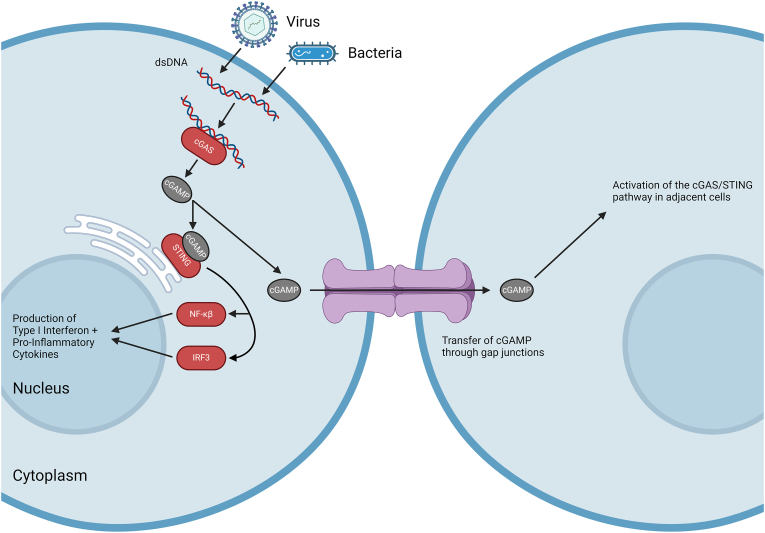
**The role of gap junctions in the cGAS/STING Pathway.** Sources of cytoplasmic dsDNA include bacteria, genomes of dsDNA viruses and DNA produced by reverse transcription of retroviral genomes. Other sources of dsDNA include dying cells and mitochondrial DNA (not shown). Binding of cytoplasmic dsDNA to cGAS leads to the generation of cyclic GMP-AMP (cGAMP) which is recognised by stimulator of interferon genes (STING). STING is shown on the endoplasmic reticulum membrane but also exists on the cell membrane and in the nucleus of cells. Through gap junctions, cGAMP is able to be passed to adjacent cells, allowing activation of the cGAS/STING pathway in cells without cytoplasmic dsDNA. Once bound by cGAMP, STING activates factors such as NF-кB and IRF3, which translocate to the nucleus to stimulate the production of pro-inflammatory cytokines and type I interferons. These responses lead to the production of factors which restrict pathogens through various mechanisms such as by inhibiting viral replication. *Created in BioRender. Scott, H. (2024) BioRender.com/n18f996*.

Importantly, connexin hemichannels and gap junctions significantly contribute to inflammation and innate/adaptive immunity, with several reviews covering this topic in depth [[24], [25], [26]]. Inflammation is a complex process that can result from the sensing of specific damage associated molecular patterns (DAMPs) or pathogen associated molecular patterns (PAMPs) which are released by damaged/infected cells. The sensing of these DAMPs and PAMPs by pattern recognition receptors (such as retinoic acid-inducible gene 1-(RIG-1) like receptors) of other resident cells leads to induction of the innate immune response [27]. Of note, connexin hemichannels and gap junctions are typically oppositely regulated in inflammation, with hemichannels opening while GJIC is reduced [28]. Cx43 hemichannels are widely reported to participate in the inflammatory response through the release of previously mentioned proinflammatory factors such as NAD+ and ATP into the extracellular space (Fig. 1). Release of ATP activates purinergic receptors, causing several downstream effects including recruitment of leukocytes to the affected area and subsequent killing of intracellular pathogens. When considering the sensing of intracellular DNA-based viruses and bacteria, Cx43 gap junctions are crucial for activation of the cGAS/STING pathway through their ability to transfer cyclic GMP-AMP (cGAMP) to adjacent macrophages. cGAMP is produced as a secondary messenger by cyclic GMP-AMP synthase (cGAS) in response to sensing of cytoplasmic DNA, which is usually derived from invading pathogens (Fig. 2). cGAMP then binds to stimulator of interferon genes (STING) which promotes the expression of factors such as interferon beta and causes subsequent amplification of the immune response [29]. Furthermore, Cx43 has also been implicated in the transfer of immunogenic peptides between cells through gap junctions. This process is critical for immune recognition of intracellular pathogens through presentation of antigens on MHC class I molecules. Transfer of these peptides from infected cells allows presentation of antigens on uninfected neighbouring cells, which can include bystander cells or professional antigen presenting cells such as dendritic cells [30]. Recognition of these peptides by cytotoxic T cells then leads to killing of the antigen-presenting cell [31].

Connexins represent an interesting target for viruses in that they can both positively and negatively influence viral infection. Due to this duality of connexins, different viruses have evolved distinct mechanisms to either disrupt hemichannel/gap junctional activity to avoid the immune response, or conversely enhance hemichannel/gap junctional signalling to maximise cell-cell spread of the virus and promote cellular disruption and inflammation. Traditionally, it has been difficult to untangle the relative contributions of hemichannels and gap junctional channels to these processes due to the unspecific nature of connexin targeting compounds, though more specific inhibitors are now becoming available, for example through the development of connexin mimetic peptides [32]. One useful method of assessing hemichannel/gap junctional activity is via dye uptake/transfer assays. For example, addition of dyes such as ethidium (cationic, molecular weight = 314 Da) or 4′,6-diamidino-2-phenylindole (DAPI) (cationic, MW = 279 Da) to cell culture media can be used to measure hemichannel activity, as these dyes are small enough to be taken up by hemichannels of cells and are capable of producing a fluorescent signal once bound to DNA [33,34]. In contrast, microinjection of other fluorescent dyes such as Lucifer yellow (anionic, MW = 443 Da) into cells can be used to assay gap junctional communication as these are transferred directly from cell to cell [34,35]. Partially due to the issues in distinguishing hemichannel and gap junctional activity, research into the effects of viral infection on connexins has historically been limited to a select few viruses, despite the obvious selective advantage to be gained from viral subversion of these proteins. However, the wider importance of connexins (and particularly Cx43) in viral infection is now beginning to be appreciated, with Cx43 being shown to be targeted by a diverse range of RNA and DNA viruses, including several oncogenic viruses. This short review will focus on the ability of individual viruses to alter Cx43 activity in a positive or negative fashion.

## 2 DNA viruses

### 2.1 Human papillomaviruses (HPV)

**Fig. 3:**
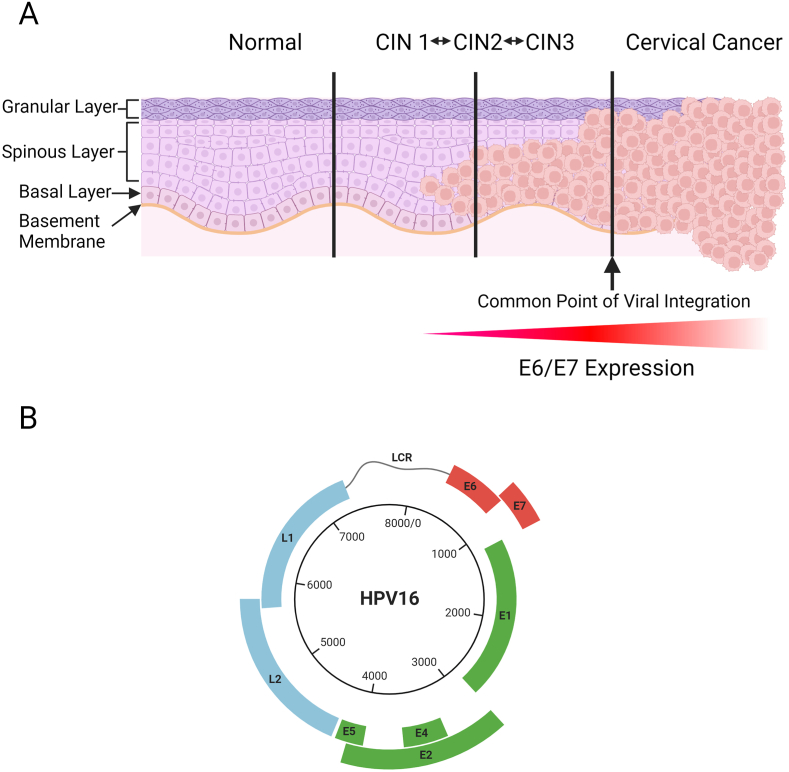
**The HPV Genome and Cervical Cancer Progression. (A)** Stages of cervical intraepithelial neoplasia (CIN) leading to cervical cancer following HPV infection. HPV infects the basal cells of the cervical epithelium through microabrasions. In these replicating basal cells, viral gene expression is low, with the productive viral lifecycle only taking place in the upper layers of the epithelium. In cells where the productive lifecycle takes place, HPV maintains cellular proliferation while inhibiting differentiation through expression of viral proteins. Infection with HPV can lead to the development of cervical intraepithelial neoplasia (CIN), which can be classified as CIN1, CIN2 or CIN3 depending on the proportion of the epithelium affected by HPV-related cell changes. The majority of CIN cases are cleared by the immune system within 24 months, however, if left untreated, a small proportion of these cases will progress to cervical cancer, which occurs when the undifferentiated persistently infected cells invade the basement membrane of the epithelium. Development of cervical cancer is heavily associated with integration of the viral genome into the host cell DNA and overexpression of the E6 and E7 oncoproteins. **(B)** The HPV16 genome is around 8 kB and encodes seven early regulatory proteins (E1, E2, E4, E5, E6, E7 and E8) and two late viral capsid proteins (L1 and L2), as well as possessing a long control region (LCR) which regulates viral transcription and replication. Upon infection of cells, the circular HPV genome is transported to the nucleus where it attaches to the host cell chromosomal DNA during cell division and is maintained in a circular (episomal) form. During cell division, the HPV genome is replicated with host cell DNA, being present in both resulting daughter cells. The HPV genome may linearise and integrate into the host cell genome following double-stranded DNA breaks, with this process resulting in a loss of the ability to produce infectious viral progeny and silencing of one or more viral genes. In the case of cancer, viral integration is also associated with unregulated expression of E6 and E7, which drive tumorigenesis. *Created in BioRender. Scott, H. (2024) BioRender.com/n15a217*.

Human papillomaviruses (HPVs) are a group of small double-stranded DNA viruses, with over 220 different genotypes described to date [36]. In a normal, transient infection HPV is present in the nuclei of cells as an episomal, or circular, 8 kb genome. Persistent infection with certain ‘high-risk’ genotypes of HPV (particularly HPV16 and HPV18) is known to be the primary cause of cervical cancer and is also responsible for a proportion of other cancers of the anogenital region, as well as up to 80 % of oropharyngeal cancers [37,38]. The development of cervical cancer is preceded by different grades of severity of cervical dysplasia, which can progress over the course of many years (Fig. 3A) [39]. However, it is important to note that, in the vast majority of cases, HPV infection is cleared by the immune system and even severe cases of cervical dysplasia can regress [40].

HPV viral genes can be divided into early (E) and late (L) genes, with the E5, E6 and E7 genes all possessing oncogenic properties (Fig. 3B) [41]. Deficiency of gap junctions has long been known to be a feature of many different types of cancer [42], including cervical cancer, with this first being noted in 1969 when electron microscopy experiments showed that adjacent cervical cancer cells often had too large a distance between their cell membranes to maintain functional gap junctions [43]. This was in contrast to normal cervical cells, where the cell membranes were in closer proximity. It has since been shown that gap junctional signalling is progressively lost over the course of cervical cancer development, with HPV-infected cell lines representing low-grade cervical dysplasia showing good gap junctional signalling (as measured by lucifer yellow dye transfer assay), while progressive derivatives of these cell lines representing a more transformed phenotype showed a gradual loss of gap junctional signalling [44]. In comparison, HPV + ve tumour cell lines, such as HeLa cells were found to have no appreciable gap junctional communication, as has been demonstrated previously [45]. Transfection of plasmids expressing fluorescently tagged Cx43 or Cx26 proteins in HeLa cells or two other cervical cancer cell lines (CaSki and SiHa) restored dye transfer, showing that the loss of gap junctional communication in these cells was due to a lack of functional connexin proteins [44]. In a separate study, transfection of HeLa cells with Cx43 was shown to reduce cell proliferation and cell cycle traverse rates, indicating Cx43 can partially restore growth control in a cervical cancer cell line [46]. Moreover, immunofluorescence experiments using sections of normal and dysplastic cervical epithelium revealed that less Cx43 protein is present in lesions representing low-grade dysplasia (CIN1/CIN2) and high-grade dysplasia (CIN3) compared to normal cervical epithelium. This effect was especially clear in high-grade lesions, where little Cx43 was observed, providing evidence that Cx43 is gradually lost during cervical cancer progression [47].

Research into the potential mechanisms behind the loss of gap junctional signalling in cervical cancer has identified Cx43 as a target of both the E5 and E6 proteins [[48], [49], [50]]. In the case of E5, transfection of HaCaT cells, a spontaneously immortalised keratinocyte cell line [51], with a plasmid expressing HPV16 E5 resulted in an 80 % decrease in lucifer yellow dye transfer between cells and reduced Cx43 protein levels in raft cultures, which are a 3D-model involving culture of epithelial cells on a collagen matrix containing fibroblasts [48,49]. These models allow for full epithelial cell differentiation and therefore more closely mimic conditions of the epithelium *in vivo* [52]. The authors also noted a clear decrease in Cx43 phosphorylation [48,49]. Phosphorylation of Cx43 at certain sites by kinases such as protein kinase A (PKA), protein kinase C (PKC) and casein kinase 1 (CK1) is known to promote gap junction assembly [53,54], which suggests that the prevention of Cx43 phosphorylation by E5 leads to the reduction in gap junctional communication. As these experiments involved transfection of plasmid containing the sequence for HPV E5, it is worth noting that E5 protein was overexpressed in these cells relative to the very small amounts of E5 protein produced during natural HPV infection, especially following integration of the HPV genome into the host cell genome, where E5 expression is often entirely absent [55]. Therefore, the observed changes in Cx43 levels caused by E5 require verification during a natural HPV infection.

While not an absolute requirement for the development of cervical cancer, integration of the episomal HPV genome into the host genome is reported in around 90 % of cases, suggesting it is an important step in tumorigenesis [56]. The event of viral integration results in the silencing of the majority of the viral genes, however, the major oncogenic proteins, E6 and E7, are instead overexpressed in cells where integration has occurred. This leads to degradation of their respective targets of p53 and pRb, which regulate apoptosis and progression through the cell cycle, resulting in uncontrolled proliferation of infected cells [57,58]. Beyond these well-defined targets, the interactions of E6 and E7 with other proteins are also crucial for the development of cancer [41]. An example of this is the interaction of E6 with PDZ (post-synaptic density protein of 95kDa/*Drosophila* Discs large tumour suppressor/Zonula occludens-1) domain-containing proteins through its C-terminal PDZ-binding motif [[59], [60], [61]]. This motif is well-conserved between high-risk HPV subtypes and is believed to be a major contributor to the tumorigenic functions of E6, with mutation of this region significantly inhibiting the ability of E6 to transform epithelial cells [62,63].

One of the PDZ proteins targeted by E6 is Dlg1, which is a member of the membrane associated guanylate kinase (MAGUK) family of proteins [64]. In addition to its roles in maintaining cell polarity and acting as a scaffolding protein, Dlg1 has also been shown to have tumour suppressor functions through its interactions with adenomatous polyposis coli (APC) and phosphatase and tensin homologue deleted on chromosome 10 (PTEN) [65,66]. The importance of Dlg1 as a tumour suppressor is further demonstrated by its targeting by at least three different oncogenic viruses; adenovirus type 9, human T-cell leukaemia virus-1 and HPV [67]. Interaction of the PDZ-binding motif of E6 with the PDZ domain(s) of Dlg1 targets it for degradation via the proteasome [60,61], with HPV18 E6 protein causing enhanced degradation of Dlg1 compared to HPV16 E6 [59].

The degradation of Dlg1 by HPV16 E6 is relevant to the loss of gap junctional communication in cervical cancer, as the N- and C-termini of Dlg1 have been shown to interact directly with the Cx43 C-terminus, providing E6 with the ability to target Cx43 through Dlg1. Early evidence for this came from HPV16+ve cervical tumour cell lines, where Cx43 protein levels and gap junctional signalling were reduced, with the majority of the remaining Cx43 protein relocating from the cell membrane to the cytoplasm, where it colocalised with Dlg1 and E6 [16]. This differed from the parental non-tumour cell line where Cx43 was localised at the plasma membrane and there was efficient gap junctional communication. As there was partial colocalisation with a marker of the lysosomes and siRNA knockdown of Dlg1 further lowered Cx43 levels in cervical cancer cells, it was proposed that Dlg1 may maintain a pool of Cx43 in the cytoplasm and protect it from degradation. This may be beneficial for cancer cells undergoing angiogenesis and metastasis, where cell-cell contacts are frequently restored in order to promote invasion, intravasation and extravasation [3].

Dlg1 does not appear to perform a similar role in non-tumour epithelial cells, where Cx43 and Dlg1 colocalise at sites of cell-cell contact but show little colocalisation in the cytoplasm [68]. In these cells, Dlg1 controls Cx43 plasma membrane localisation and gap junctional signalling, with knockdown of Dlg1 resulting in decreased Cx43 protein levels (with no change to Cx43 mRNA levels) and Cx43 being mainly located in the cytoplasm, suggesting Dlg1 is either involved in the trafficking of Cx43 to the plasma membrane or stabilisation of Cx43 at the membrane. This raises the possibility that Dlg1 performs different roles in non-tumour and tumour cells, potentially demonstrating how HPV can alter the functions of cellular proteins to promote tumour progression and metastasis. Importantly, transfection of an HPV-ve cervical cancer cell line with a plasmid expressing HPV16 E6 showed a similar effect to cells harbouring the full HPV16 genome, with decreased Cx43 protein levels and cytoplasmic localisation of the remaining protein being reported [50]. Further immunoprecipitation experiments showed that Cx43 forms a complex with Dlg1 and E6 in HPV + ve tumour cells, while siRNA depletion of E6 restored Cx43 and Dlg1 at the cell membrane. Finally, expression of E6 protein with a mutated PDZ-binding motif to block binding to Dlg1 resulted in redistribution of Cx43 to the plasma membrane, showing that the reduction in gap junctional signalling was likely partially reliant on the ability of E6 to interact with Dlg1 [50]. Despite this, the lack of a complete reversal of the effect caused by E6 in these cells may point to additional mechanisms by which E6 disrupts Cx43, with some preliminary evidence pointing to the possibility of a direct interaction between E6 and Cx43 (Graham group, unpublished data).

It is important to note that although normal immortalised keratinocytes (NIKS) transfected with the HPV18 genome have lower levels of Cx43, gap junctional intercellular communication is conversely increased compared to uninfected cells due to an upregulation of Cx45 [69]. This suggests other changes associated with cervical cancer progression in addition to E6 expression are necessary to impair gap junctional communication.

Regardless of whether HPV targets Cx43 directly or indirectly, depletion of Cx43 offers several advantages to the virus. Firstly, Cx43 can act as a tumour suppressor protein (see Aasen et al., 2019 for review), including by directly interacting with and sequestering β-catenin at the cell membrane and preventing its translocation to the nucleus to promote cell proliferation via the Wnt signalling pathway [21,70]. Wnt signalling is activated by HPV E6, a feature which is abrogated if the PDZ-binding motif is truncated [71]. Therefore, one of the ways in which E6 may act to promote activation of the Wnt pathway (and therefore cellular proliferation) is through degradation/translocation of Dlg1, which would cause altered localisation and a reduction in Cx43 levels, allowing free β-catenin to translocate to the nucleus. Secondly, the removal of Cx43 from the plasma membrane inhibits activation of the immune response through hemichannel activity and gap junctional sharing of immune factors, allowing HPV to evade the immune system and continue to replicate in infected cells.

### 2.2 Human adenovirus type 5 (HAdV-5)

As a further member of the small DNA tumour virus group, adenoviruses are also non-enveloped double-stranded DNA viruses. This group is diverse, with at least 67 different serotypes which are associated with mostly self-limiting infections of the gastrointestinal tract, the respiratory tract and the eye [72]. On the other hand, adenovirus infection can also be associated with more severe disease, including viral myocarditis, which can be fatal [73]. Adenovirus infection is also of particular concern in the case of immunocompromised patients, where infection is associated with hepatitis, pneumonia and meningoencephalitis [74]. While adenoviruses have been convincingly shown to cause cancer in a variety of animal models [75,76] and can transform human cell lines *in vitro* [77], they have never been demonstrated to cause cancer in humans. Despite this, they share several features with HPV and other cancer-causing viruses, including the ability to target p53 and pRb [78].

As with HPV, human adenovirus type 5 (HAdV-5) has also been shown to target Cx43, with this effect likely being important in the context of viral myocarditis [79,80]. Infection of HaCaT cells with HAdV-5 resulted in dramatically lower levels of Cx43 by Western blot, with these being reduced by up to 95 % at 72 h post-infection compared to 0 h post-infection. This was concordant with a significant decrease in the ability of these cells to transfer lucifer yellow dye, even though the small level of remaining Cx43 protein was mainly localised to the cell membrane. Two separate mechanisms were found to contribute to the reduction in Cx43 protein levels and gap junctional signalling. The first of these was due to levels of Cx43 mRNA being reduced by over 50 % in these cells at the 24 h post-infection timepoint. As a regulator of the transcription of the *GJA1* gene which is increased in adenoviral infection, the authors next investigated whether β-catenin was involved in the transcriptional repression of Cx43 in infected cells. Blocking of β-catenin activity through use of an inhibitor (LF3) resulted in partial restoration of *GJA1* mRNA levels, suggesting that β-catenin was important for suppression of Cx43 expression in these cells [79]. This was an interesting finding given that β-catenin has previously been reported as a transcriptional activator of *GJA1* [81], emphasising that viral infection can drastically alter the functions of cellular proteins and their interaction partners, as may be the case for the role of the interaction between Cx43 and Dlg1 during HPV infection. Further experiments revealed that the HAdV5 early protein E4 open reading frame 1 (E4ORF1) was sufficient for activation of β-catenin, leading to the repression of *GJA1* expression. The second mechanism of Cx43 gap junctional signalling disruption was through phosphorylation of Cx43 at residue S373 by protein kinase B (AKT) [79], which is associated with sequential phosphorylation of S279/S282 and S368 (which was also found to be increased), causing initial growth of gap junction plaques before subsequent internalisation of Cx43 [53].Crucially, the ability of HAdV-5 to target Cx43 appears to be conserved in human cardiomyocytes derived from induced pluripotent stem cells, as Cx43 protein levels and gap junctional communication are reduced in these cells following infection [80]. Building on previous data, the authors showed that the increase in phosphorylation at site S368 was also conserved in these cells, additionally showing that viral activation of PKC was responsible for this change, as blocking PKC activity through two different inhibitors (sotrastaurin and bisindolylmaleimide) abrogated this effect. Furthermore, mutation of the Cx43 S368 residue to an alanine residue, which is unable to be phosphorylated, was able to prevent cardiac conduction slowing caused by HAdV-5, suggesting that the targeting of Cx43 by HAdV-5 may be a driving factor behind subsequent development of viral myocarditis [80]. Taken together, these data suggest that HAdV-5 is capable of targeting Cx43 and gap junctional communication through several different mechanisms, likely contributing to cardiac arrhythmogenesis during acute infection.

### 2.3 Other oncogenic DNA viruses

Currently, very few studies exist on the effect of human oncogenic viruses other than HPV on Cx43. Despite this, there are some indications that Cx43 is altered in cancers caused by different oncogenic viruses, though whether these effects are directly caused by viral factors or are brought about by more general cellular changes during tumorigenesis is currently unknown. For example, Cx43 expression is often increased in hepatocellular carcinoma (HCC) [82,83], which, in the majority of cases, arises from chronic infection with the hepatitis B virus (HBV, DNA virus) or hepatitis C virus (HCV, RNA virus) [84]. Supporting this, a study by Leroy and colleagues found that levels of Cx43 mRNA and protein were significantly increased in human HCC cell lines relative to primary human hepatocytes [85]. Interestingly, Cx43 levels also appeared to be increased in HBV-positive HCC cell lines relative to HBV-negative HCC cell lines, raising the possibility that HBV infection may promote Cx43 expression. Despite this, there is conflicting evidence for the role of Cx43 in HCC. Some studies suggest Cx43 expression promotes growth, invasion and metastasis in HCC [83,86], while in the context of HBV-positive HCC, Cx43 positivity by histopathology was found to be associated with both better prognosis and later recurrence in certain cohorts when compared to Cx43-negative HCC samples [87]. Future studies are needed to address whether alterations in Cx43 in HCC can be caused by HBV (or HCV) viral factors.

Merkel cell polyomavirus (MCPyV) is a recently discovered double stranded DNA tumour virus which is associated with the development of Merkel Cell Carcinoma (MCC), a rare cancer with a high mortality rate. MCPyV is present in approximately 75 % of MCC cases [88]. Only one robust study to date has investigated Cx43 in MCC [89], including a total of 32 MCC cases, with 18 of these being MCPyV positive. Using immunohistochemistry, the authors showed that both MCPyV positive and negative cases had very low levels of Cx43, indicating that loss of Cx43 is associated with MCC. Interestingly, transformation of human trophoblasts using the T antigen of simian virus 40 (SV40), a different polyomavirus which causes tumours in animals but has not conclusively been shown to cause human cancer [90], resulted in reduced Cx43 expression and protein levels in these premalignant cells relative to normal cells [91]. Further evidence of polyomaviruses potentially regulating Cx43 comes from a transgenic mouse model expressing the polyomavirus middle T antigen, resulting in mammary tumour formation and defects in gap junctional communication [92]. Treatment of these mice with a quinoline derivative called PQ1 (which has been shown to promote gap junctional signalling in tumour cells) [93] resulted in increased Cx43 protein levels in the early stages of tumour formation, significantly inhibiting tumour growth at all stages of tumour development [94]. Taken together, these data may point to conserved targeting of Cx43 among polyomaviruses, however further studies are required to prove this. In contrast to this, a study including 15 cases of Kaposi sarcoma, a cancer caused by human herpesvirus 8 (HHV8), found that Cx43 was mislocalised and resided predominantly in the cytoplasm of tumour cells rather than at the cell membrane [95]. These early results indicate that infection with other oncogenic viruses can lead to changes in Cx43. Future studies of these oncogenic viruses will be instrumental in proving whether Cx43 is specifically targeted by viral factors to promote cancer progression.

## 3 Retroviruses

### 3.1 Human immunodeficiency Virus-1 (HIV-1)

Unlike most of the viruses discussed up to this point, human immunodeficiency virus (HIV) does not directly contribute to the development of human cancers. Despite this, HIV infection increases risk of developing many types of cancer, including those caused by oncogenic viruses detailed above such as HPV and HHV8, meaning HIV research is highly relevant to the tumour virus field.

A member of the *Retroviridae* family, HIV is a single-stranded positive-sense RNA virus that infects CD4 helper T-cells and macrophages, leading to the development of acquired immunodeficiency syndrome (AIDS). Following infection, HIV reaches the central nervous system, where it can cause a variety of symptoms including neurocognitive impairment and HIV-associated dementia, summarised under the banner of HIV-associated neurocognitive disorders (HAND) [96]. Despite the existence of effective treatments to suppress replication of the virus, allowing HIV patients to live a near-normal life, HAND is still present in many patients and remains an important issue. This is because the development of HAND is associated with increased inflammation rather than active viral replication, a feature that prompted researchers to study the role of connexins in HIV infection.

Early evidence for a role of gap junctions in the neuropathology of HIV infection came from the discovery that HIV-infected astrocytes were capable of transmitting toxic signals to neighbouring uninfected cells, causing apoptosis, while infected cells themselves were resistant to apoptosis [97]. Use of general gap junction blockers such as octanol or α-glycyrrhetinic acid (AGA) [98] drastically reduced this effect, suggesting that the induction of apoptosis in HIV-infected adjacent cells was reliant on gap junctional signalling [97]. The authors also noted that Cx43 protein levels remained unchanged in infected cells, implying that HIV maintained Cx43 in order to preserve communication with neighbouring cells. Through immunofluorescence experiments, a further study confirmed that Cx43 is actually upregulated in HIV-infected astrocytes and that this effect is caused by the HIV-tat protein, which is a transcriptional regulator for the virus, while other tested HIV proteins (Vif, Gag, Rev, Nef) had either no effect on Cx43 protein levels or slightly reduced Cx43 levels (gp120) [99]. As a controller of viral and cellular gene expression [100], the increase in Cx43 levels in HIV-tat treated cells was expected to be due to an increase in Cx43 mRNA levels, with qRT-PCR experiments confirming a ∼6-fold increase in Cx43 mRNA levels in astrocytes upon treatment with HIV-tat. Finally, the use of chromatin immunoprecipitation showed that HIV-tat was capable of binding to the Cx43 promoter region, suggesting that this mechanism was responsible for the upregulation of Cx43 in these cells [99].

In addition to tat, the HIV envelope glycoprotein, gp120, has also been shown to cause changes in Cx43 in astrocytes [101]. Treatment of cultured human astrocytes with gp120 (which can be secreted by infected cells during infection) resulted in an increased rate of ethidium uptake, while inhibition of Cx43 channels by preincubation with two different Cx43 mimetic peptides (Tat-L2 or gap19) blocked this effect, suggesting gp120 triggered an increase in hemichannel activity in these cells. Cx43 hemichannel activity has been reported to be elevated in several neuropsychiatric disorders such as Alzheimer's disease [102], indicating that the development of HAND may be related to the modulation of Cx43 activity by HIV proteins. Interestingly, gp120 treatment did not affect Cx43 localisation or cell-cell coupling, as transfer of microinjected ethidium between cells was uninhibited by gp120 treatment (this experiment used La^3+^ to block transfer of ethidium through hemichannels). This suggests that gp120 specifically augments Cx43 hemichannel activity without altering Cx43 gap junctional communication.

While HIV infection increases hemichannel activity and maintains gap junctions in astrocytes, it is also known to promote the formation of tunnelling nanotubes (TNTs) in macrophages [103]. A relatively new discovery, TNTs are long and thin actin-based membranous protrusions which can mediate longer-range communication between cells. TNTs allow cells to exchange both smaller and larger items, even allowing the transfer of whole organelles such as mitochondria [104]. Recently, Cx43 has been shown to be involved in gap junctional signalling at the tip of TNTs [105], which may be involved in cancer pathogenesis by influencing tumour invasion, proliferation and treatment resistance [106,107]. Similarly, Cx43 is localised at the base and tips of TNTs in HIV-infected macrophages, while uninfected macrophages did not appear to express Cx43 [108]. Lucifer yellow dye injection experiments showed that Cx43 gap junctions at TNT tips were functional and could pass the dye to other cells, while addition of the same dye to the cell medium resulted in no dye uptake, suggesting hemichannels were not active in these cells. Furthermore, addition of AGA as a gap junction blocking compound did not prevent TNT formation but did remove the ability of microinjected dye to spread between infected cells with TNTs, providing evidence that infection with HIV specifically results in upregulation of Cx43 and gap junctional communication in these cells, with no notable hemichannel activity. Due to the suspected ability of HIV to directly infect other cells through TNTs [103], the authors next investigated whether this process was dependent on Cx43 gap junctional communication. Both the formation of TNTs and the normal functioning of Cx43-containing gap junctions were found to be required for efficient cell-cell spread of the virus, while application of a gap junction blocker and virus simultaneously in these cells additionally showed an inhibition of viral replication [108]. This implicates Cx43 in the early stages of initial infection with HIV, allowing the virus to spread with high efficiency between cells in order to establish infection and evade the immune system [109]. In addition, Cx43 also likely contributes to the ability of HIV to rapidly reestablish populations of infected cells from the latent reservoir following interruption of treatment, as this process requires efficient cell-cell spread given the low starting viral load [108].

Taken together, these data suggest that HIV infection can modulate Cx43 differentially in various cell types, causing an increase in hemichannel activity in astrocytes and an upregulation of Cx43 gap junctions in TNTs in macrophages. These mechanisms appear to be crucial in various different aspects of the HIV lifecycle and pathogenesis, including viral replication and cell-cell spread, as well as in the transport of toxic factors to adjacent uninfected cells, exemplifying how viruses can subvert Cx43 to suit their own needs.

### 3.2 Human T-cell lymphotropic virus Type-1 (HTLV-1)

While HIV is the most well-documented retrovirus in terms of modulating Cx43, a further retrovirus, human T-cell lymphotropic virus type-1 (HTLV-1), has also been shown to promote the formation of gap junctions [110]. HTLV-1 is a directly-acting oncogenic virus which causes adult T-cell leukaemia/lymphoma, as well as a debilitating neurological disease called HTLV-associated myelopathy [111]. In an *in vitro* study, gap junctional communication was found to be increased between HTLV-1 transformed T-cells and uninfected endothelial cells (compared to communication between uninfected transformed lymphocytes and endothelial cells), with this effect likely contributing to the ability of transformed cells to invade normal tissue and promote metastasis [110]. Building on this work, Bazarbachi and colleagues investigated whether the HTLV-1 viral oncoprotein Tax could be involved in promoting gap junctional communication through Cx43 [112]. Co-transfection of HeLa cells with Tax and a reporter plasmid containing the sequence of luciferase under the control of the Cx43 promoter region revealed that Tax expression induced a 6-fold increase in Cx43 promoter activity, as well as promoting formation of gap junctions with endothelial cells. These results suggest that through upregulation of Cx43 caused by Tax, HTLV-1 establishes functional gap junctions with other cell types in order to promote invasion.

## 4 RNA viruses

### 4.1 Severe acute respiratory syndrome coronavirus 2 (SARS-CoV-2)

While this review has focused primarily on viruses associated with human cancers, severe acute respiratory coronavirus 2 (SARS-CoV-2) continues to be highly relevant in the virology field. The following section summarises the existing work on regulation of Cx43 by SARS-CoV-2, serving as an important example of how viruses from diverse backgrounds can target Cx43 in similar ways.

**Fig. 4:**
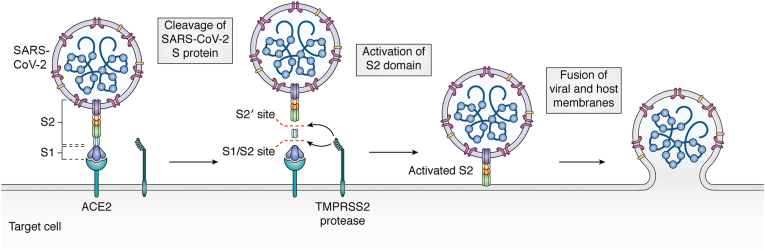
**Entry Route of SARS-CoV-2 into susceptible cells.** The Spike protein of SARS-CoV-2 is composed of the S1 and S2 subunits, with S1 binding to the angiotensin converting enzyme 2 (ACE2) receptor, while the S2 subunit mediates fusion with the host membrane. On binding of S1 to ACE2, the TMPRSS2 cellular protease cleaves the spike protein at both the S1/S2 site and the S2′ site. This activates fusion activity of the S2 domain, which then allows fusion of the membranes, resulting in internalisation of the viral genome and the ACE2 receptor. Figure from Hartenian et al., 2020 [120].

SARS-CoV-2 is a positive-sense, single-stranded RNA virus belonging to the *β-coronavirus* genus. Following its first detection in 2019, the virus has since spread rapidly, causing a global pandemic and around 7 million deaths to date [113]. SARS-CoV-2 is known to primarily cause acute respiratory illness but can also result in the development of long COVID, a poorly understood chronic illness with widespread symptoms including fatigue and anosmia, as well as ‘brain fog’ and heart palpitations [114]. SARS-CoV-2 possesses 4 structural proteins: spike (S), membrane (M), envelope (E) and nucleocapsid (N) [115]. The spike protein of SARS-CoV-2 on the surface of the virion is essential for infection of susceptible cells through interaction with the angiotensin converting enzyme 2 (ACE2) receptor (Fig. 4). Spike protein is composed of two subunits: S1 and S2, with the S1 subunit being responsible for binding of the ACE2 receptor, while S2 controls viral envelope fusion with the host cell membrane, resulting in internalisation of the virus and the interacting receptor [116]. Additionally, the spike protein has been implicated in endothelial cell dysfunction and inflammation, one of the factors associated with both acute respiratory disease and long COVID [[117], [118], [119]].

Primarily due to its known role in the promotion of inflammation, several recent studies have investigated the potential modulation of Cx43 by the SARS-CoV-2 spike protein. The first of these studies showed that a 12 h treatment of primary mouse brain endothelial cells with 10 μg/ml S1 protein resulted in a dramatic 80 % decrease in total Cx43 protein levels, with a similar loss of Cx43 being reported in mouse cerebral arteries treated with S1 [121]. While subcellular localisation of Cx43 was not directly investigated in this study, the decrease in Cx43 protein levels was coincident with a ∼2-fold increase in the association of Cx43 with Rab5a, a protein which regulates fusion of early endocytic vesicles and early endosomes. The association of ACE2 and Rab5a was found to be similarly increased, suggesting that S1 treatment promotes endocytosis of both ACE2 and Cx43, which could feasibly lead to their subsequent degradation through the lysosomal pathway as occurs during the regular Cx43 lifecycle [122]. Interestingly, untreated diabetic mouse cells were found to possess lower levels of Cx43 protein relative to non-diabetic cells, with these levels being further reduced following S1 treatment [121]. This indicates that these cells were at an increased risk of loss of gap junctional communication on exposure to S1, demonstrating how preexisting health conditions can compound the effects of viral infection. These findings contrast with work carried out by Freda et al., which investigated the effect of short-term exposure (3 μg/ml for 1 h) of SARS-CoV-2 S, M or N proteins on Cx43 levels in human umbilical vein endothelial cells. In these experiments, Cx43 was found to be significantly increased on the surface of cells following treatment with S or M protein, but not N protein, suggesting the spike/membrane proteins may conversely enhance Cx43 cell membrane localisation [123].

In a further study, the ability of S1 protein to augment Cx43 hemichannel and gap junctional activity was investigated using HeLa cells. These experiments used cells transfected with fluorescently tagged Cx43, as standard HeLa cells are deficient in gap junctions and are therefore not well-coupled [45]. Treatment with S1 protein was found to increase the uptake of ethidium dye in transfected cells, representing an increase in Cx43 hemichannel activity [124]. The increase in dye uptake of Cx43 transfected cells following S1 treatment was dose-dependent and was also enhanced in HeLa cells transfected with both Cx43-GFP and ACE2-mCherry. In these cells, Cx43 and ACE2 both localised to the plasma membrane, with ACE2 additionally showing some cytoplasmic localisation. This result suggests the presence of ACE2 enhances the ability of spike protein to increase Cx43 hemichannel activity. The authors additionally assessed gap junctional communication in these cells via a scrape loading dye transfer assay, where a 55 % decrease in dye transfer was observed following treatment with S1 protein compared to control cells, suggesting that the increase in Cx43 hemichannel activity in spike-treated cells is concordant with a loss of gap junctional intercellular signalling [124]. Overall, this pattern of hemichannel and gap junctional activity is similar to that reported for cells in an inflammatory environment [125].

From the currently available data, it is difficult to conclude whether SARS-CoV-2 (particularly the spike protein) positively or negatively regulates Cx43. It is also feasible that SARS-CoV-2 could both positively and negatively regulate Cx43 under different circumstances. Despite this idea, there are several differences between the existing studies which could account for the contrasting findings, including the use of the full length spike protein as opposed to the S1 subunit, concentration of spike/S1 protein, treatment time, type of cells used and method of measurement. Interestingly, two further β-coronaviruses, human OC43 and murine hepatitis virus, have also been shown to modulate Cx43. OC43 causes a common cold and infection of A549 cells, a lung carcinoma cell line, resulted in an inhibition of both hemichannel activity and gap junctional communication through a reduction of Cx43 protein levels and impaired Cx43 trafficking to the plasma membrane [126]. In addition, mouse hepatitis virus has been found to cause a similar effect in primary astrocytes/meningeal fibroblasts and *in vivo* in mouse brain tissue [127,128]. This raises the possibility that certain β-coronaviruses may down-regulate Cx43 activity through a conserved mechanism, perhaps to disrupt the immune response. However, further research is required to conclusively demonstrate the exact mechanism for these changes and whether SARS-CoV-2 regulates Cx43 in a similar fashion or using a novel mechanism.

## 5 Conclusion

In conclusion, an increasingly diverse array of viruses are being appreciated to target Cx43. These comprise retroviruses and RNA/DNA viruses, including viruses belonging to the small DNA tumour virus family. This diversity is reflected in the mechanisms by which these viruses target Cx43, with different viruses targeting Cx43 at the transcriptional level, the protein level and the level of post-translational modifications. This targeting can result in promotion of Cx43 signalling in some cases, while in other cases Cx43 expression and function are minimised. In addition to the viruses discussed in this review, others can also influence connexins in various ways, including several members of the *Herpesviridae* (herpes simplex virus-2, cytomegalovirus and the previously mentioned HHV8) and the *Flaviviridae* (Zika virus) [[129], [130], [131]]. In the case of herpes simplex virus-2, infection of Vero cells (a monkey kidney cell line) resulted in reduced gap junctional communication [129], however the effect of the virus on individual connexin levels remains to be investigated. In contrast, cytomegalovirus is known to specifically target Cx43 by the viral immediate early (IE) proteins IE72 and IE86, promoting degradation of Cx43 through the proteasome and reducing overall Cx43 levels in human glioblastoma multiforme cells [130]. Similarly, Zika virus mediates degradation of Cx43 through the proteasome in mouse cardiomyocytes, though a specific viral factor is yet to be identified for this effect [131]. This process may be involved in the ability of Zika virus to cause cardiovascular dysfunction, as with adenovirus type 5 discussed above [79]. The ability of both an RNA and a DNA virus to target Cx43 for degradation via the proteasome highlights that diverse viruses may are be able to use similar pathways to regulate Cx43. A summary of the viruses discussed in this review and their effects on Cx43 is shown in Table 1. Given the critical functions of Cx43 and the advantages to be gained from viral subversion of this protein, other viruses are sure to be added to this growing list with further research.

**Table 1:** Viral modulation of Cx43 and associated viral factors.

Virus	Effect on Cx43	Viral Factor (if known)	Reference
**HPV**	↓Phosphorylation/GJIC	E5	[48,49]
	Cx43 relocated to the Golgi ↓PL/GJIC	E6	[16,47,50,68]
**HAdV-5**	↓ mRNA/PL/GJIC ↑ p368 phosphorylation	E4-ORF1	[79,80]
**HBV**	↑ mRNA/PL	?	[85]
**MCPyV**	↓ PL	?	[89]
**SV40**	↓ mRNA/PL/GJIC	T antigen	[91]
**HHV8**	Cx43 relocated to the cytoplasm	?	[95]
**CMV**	↓ PL/GJIC	IE72, IE86	[130]
**SARS-CoV-2**	↓ PL	S protein	[121]
	↑ HA ↓ GJIC	S protein	[124]
	↑ Cell membrane expression	S, M proteins	[123]
**OC43**	↓ PL/HA/GJIC	?	[126]
**MHV**	Cx43 relocated to the ER ↓ PL/GJIC	?	[127,128]
**HIV-1**	↑ mRNA/PL	tat	[99]
	↑ HA	gp120	[101]
**HTLV-1**	↑ mRNA/GJIC	Tax	[110,112]
**ZIKV**	↓ PL	?	[131]

GJIC=Gap junctional intercellular communication, HA=Hemichannel activity, PL=Protein levels.

## CRediT authorship contribution statement

**Harry Scott:** Writing – review & editing, Writing – original draft, Visualization, Conceptualization. **Patricia E. Martin:** Writing – review & editing, Supervision, Funding acquisition. **Sheila V. Graham:** Writing – review & editing, Visualization, Supervision, Funding acquisition, Conceptualization.

## Funding

The Cx43 work carried out by HS in the Graham and Martin laboratories was funded by a grant (005_S_19) from the 10.13039/501100000296British Skin Foundation.

## Declaration of competing interest

The authors declare that they have no known competing financial interests or personal relationships that could have appeared to influence the work reported in this paper.

## Data Availability

No data was used for the research described in the article.
